# Some Points in the Midwives Bill

**Published:** 1895-05-18

**Authors:** 


### The Hospital

An Institutional Journal of

The Medical Sciences and Hospital Administration, with

AN EXTRA NURSING SUPPLEMENT.

Vol. XVIII.—No. 451.] [May 18, 1895.

### Some Points in the Midwives Bill.

It is, in our judgment, right and reasonable that any statutory act for the registration of midwives shall consist of such provisions, and of such only, as shall be satisfactory to the right-minded members of the medical profession. The medical profession is, or ought to be, the health providence, the State director of hygiene, for all ranks of the people. If this be so, and it is, then clearly any Act of Parliament relating to any question of health which is displeasing to the majority of fair-minded medical men is *ipso facto* condemned.

A Bill for the compulsory registration of midwives has been introduced to the House of Lords by Lord Balfour of Burleigh, and was read a second time, with the approval of the Government, on 14th inst If the Bill in its main provisions be such as a medical man who wishes to "do as he would be done by" can approve, it is for that medical man, in his capacity of health conservator for the commonwealth, to give it his loyal and determined support. Unfortunately, determination is not a characteristic of some members of our profession. Their attitude is too frequently that known in political circles as "sitting on the fence." There is no necessity for sitting on the fence as regards the main principles of the Midwives Bill. Medical men may on the one hand "do as they would be done by," and on the other they may act in the narrowest spirit of a mistaken self-interest, and so bring obloquy upon themselves and our whole profession. Many will do the former, and, perhaps, a few the latter. Let us hope the few will be very few.

The essential provisions of the new Bill are few in number and of the most perfect simplicity. The least experienced member of our profession may understand them quite well without any outside help, either of a sophisticated or of an unsophisticated nature. It is our hope that medical men, especially the younger members of the profession, will trust their own judgment in this matter, and refuse absolutely to be led by agitators.

What, in the first place, is a "midwife," according to the definition of the new Bill? She is "a woman who undertakes to attend cases of natural labour." Now nothing can be more certain than this, that an adequately trained woman is a fit and proper person to attend cases of "natural labour." It is believed that no fewer than 450,000 cases of childbirth are attended annually by midwives in England and Wales. These women are attended and will continue to be attended by midwives. Is it better that they should be attended by "trained" or by "untrained" midwives? Ought the medical profession, as the providence and conservator of the public health, to support training or no training? We do not hesitate to say that if no financial interests were involved, or supposed to be involved, there are not a dozen men in the whole profession who would stand for no training.

We have considered what a registered midwife may do under the provisions of the Bill. She may attend "natural labours." Now let us consider what she may not do. "A certificate under this Act," runs the Bill, "shall not confer upon any woman any right or title to be registered under the Medical Act, 1858, or the Acts amending the same, in respect of such certificate, or to assume any name, title, or designation implying that she is by law recognised as a licentiate or practitioner in medicine or surgery, or that she is qualified to grant a certificate of death or of stillbirth." Here is a provision, as strict as words can make it, for the preventing of registered midwives from encroaching in any degree whatever upon ordinary medical practice, for which, of course, they will have no training and no competence. In other words, midwives are to be midwives and nothing else. They are midwives now, only they are untrained; they will continue to be midwives if the Bill becomes law; but they will be trained, registered, and fitted for their work.

The third point of importance to the members of our profession in the new Bill is that midwives are to be trained, registered, and supervised in their life work entirely by the medical profession. The "Midwives Board" of education, registration, and administration "shall consist of twelve registered medical practitioners." Substantially these three provisions constitute the new Bill. Of course there are details, but none which contravene in any way whatever these three main principles. To repeat in a sentence what has been said: the midwife, under the new Bill, will attend natural labours, and natural labours only; she will continue to be, as she is now, a midwife and nothing but a midwife; but, in the third place, there will be this important difference, that whereas now she can practice, without let or hindrance, everything and in whatever way she pleases, under the new Bill her duties will be limited and defined with the utmost possible strictness; and her absolute masters and judges will be registered medical practitioners.

This Bill, with such necessary modifications as may be suggested in Committee, ought certainly to become law. For our part we are ashamed to be told that "in England alone, of all European countries," nearly half a million of poor women are annually attended by all sorts and conditions of more or less untrained women in their hour of peril, and that the State takes no heed, whilst the medical profession is the great supporter of this ignorant, wicked, and deadly system. Is it nothing to doctors that the mortality among poor women in childbirth is needlessly high? Is it nothing that many people pass through life hopelessly blind from infantile ophthalmia, whose eyesight might be easily preserved by the smallest modicum of elementary knowledge on the part of the midwife? The medical profession is the most humane of all the professions when taken man by man. Who will believe in the sincerity of our individual humanity if, when the profession acts together as a whole, it turns a deaf ear to the cry of motherhood in the hour of its direst peril? There is no other honourable course before us but to do our plain duty in this matter, be the consequences what they may.

